# Abnormal Trophoblast Invasion in Early-Onset Preeclampsia: The Involvement of Cystathionine β-Synthase, Specificity Protein 1 and microRNA-22

**DOI:** 10.7759/cureus.104353

**Published:** 2026-02-27

**Authors:** Pallavi Arora, Sahil Kalra, Renu Dhingra, Pallavi Kshetrapal, Neerja Bhatla, Sadanand Dwivedi

**Affiliations:** 1 Department of Anatomy, Shri Mata Vaishno Devi Institute of Medical Excellence, Katra, IND; 2 Department of Anatomy, All India Institute of Medical Sciences, New Delhi, New Delhi, IND; 3 Department of Mechanical Engineering, Indian Institute of Technology Jammu, Jammu, IND; 4 Maternal and Child Health, Translational Health Science and Technology Institute, Faridabad, IND; 5 Department of Obstetrics and Gynaecology, All India Institute of Medical Sciences, New Delhi, New Delhi, IND; 6 Department of Biostatistics, All India Institute of Medical Sciences, New Delhi, New Delhi, IND

**Keywords:** cystathionine β-synthase (cbs), early onset preeclampsia (eope), matrix metalloproteinase (mmp), specificity protein 1 (sp1), tissue inhibitor of metalloproteinase (timp), trophoblast invasion

## Abstract

Objectives: To determine and compare the expression of matrix metalloproteinases (MMPs) 2, 9, tissue inhibitors of metalloproteinase (TIMPs) 1, 2, cystathionine β-synthase (CBS), specificity protein 1 (Sp1), and microRNA-22 (miR-22) in the early-onset preeclampsia (EOPE) patients and normotensive controls at both transcriptional and translational levels.

Methods: The study was carried out on EOPE women (n=50) as cases, and normotensive, non-proteinuric pregnant women (n=50) as controls, enrolled at the Department of Obstetrics and Gynaecology. Expression/levels of MMPs 2, 9, TIMPs 1, 2, CBS, Sp1, and miR-22 were determined in venous blood. Thirty cesarean-delivered placentae (15 each of EOPE patients and controls) were collected to analyze the expression/levels of the markers in the tissue.

Results: Significantly reduced mRNA and protein expression/levels of MMPs 2, 9, CBS, and Sp1, whereas elevated levels of TIMP-1 and TIMP-2 were observed in EOPE participants as compared to controls. The gene expression of miR-22 was found to be significantly upregulated in EOPE participants compared with controls.

Conclusions: This is the first study of its kind, which implies that insufficient trophoblastic invasion may be because of downregulation of MMPs 2, 9, CBS, Sp1, and concomitant upregulation of TIMPs 1, 2, both in the placentae collected at delivery and the circulating blood collected at diagnosis. The regulation of Sp1 by miR-22 in the EOPE participants as compared to controls was also evident by the expression of miR-22 assayed in the same set of samples.

## Introduction

Preeclampsia (PE) is a multisystem disorder of pregnancy defined by high blood pressure and proteinuria [1]. The hallmark of PE is the impaired capacity of the trophoblast to invade the uterine spiral arteries, which results in a poorly perfused fetoplacental unit [2]. PE is divided into two categories based on the stage of pregnancy in which it appears: early-onset preeclampsia, diagnosed before 34 weeks of gestation, or late-onset preeclampsia, which is diagnosed at or after 34 weeks or later [3,4]. It is thought that early-onset PE poses a high risk to both mother and fetus [5], whereas late-onset PE may present with less severe clinical symptoms [6]. Impaired trophoblast invasion observed in the early stage of PE is characterized by a decrease in MMP-2 and MMP-9, which affects spiral artery remodeling, causing a dysfunctional uteroplacental circulation [7]. MMPs and their inhibitors (tissue inhibitors of metalloproteinase, TIMPs) play a significant role in trophoblast invasion into the uterine wall [7]. The identification of changes in the levels and activity of MMPs 2 and 9, as well as their endogenous inhibitors (TIMPs 2, 1) in both defective trophoblast invasion and endothelial dysfunction, led to the consideration of these proteases as critical mediators in the pathological features of preeclampsia [7]. Hydrogen sulfide (H₂S) mediates vascular resistance and systemic blood pressure, has anti-inflammatory effects, promotes angiogenesis, protects against ischemia-reperfusion injury, and has been reported to influence MMP-2 expression in vitro; treatment of hepatocellular carcinoma or hepatoma cells with sodium hydrogen sulphide (NaHS) (H₂S donor) caused up-regulation of MMP-2 protein expression, suggesting H₂S promotes invasion in various cancer cell lines [8]. Endogenously, H₂S is produced as a metabolite of homocysteine (Hcy) by cystathionine β-synthase (CBS), cystathionine γ-lyase (CSE), and 3-mercaptopyruvate sulfurtransferase (3MST) [9]. The regulation of CBS -1b and -1a promoters in a proliferation-sensitive manner by specificity protein 1 (Sp1) has already been reported [10], where transfection of Sp1-deficient fibroblasts by Sp1 expression construct showed significantly high levels of CBS expression [10]. Sp1 was identified as a novel, direct target of microRNA-22 (miR-22) using a luciferase reporter assay, in which miR-22 overexpression diminished, and its knockdown upregulated Sp1 mRNA and protein expression in colorectal carcinoma cells, suggesting an inverse relationship between miR-22 and Sp1 [11]. Interestingly, miR-22 expression was reported to be increased in early-onset preeclampsia (EOPE) placentae compared to placentae from preterm labour controls [12]. The present study aims to determine and compare the transcript and protein expression of MMPs 2, 9, their inhibitors (TIMPs 2, 1), cystathionine β-synthase, specificity protein-1, precursor miR-22, and miR-22-3p in the blood samples and placentae from the pregnancies complicated by EOPE and healthy pregnant women (normotensive, non-proteinuric). This article was previously posted to the Social Science Research Network (SSRN) preprint server on February 20, 2025 (doi: 10.2139/ssrn.5145887).

## Materials and methods

Study subjects

One hundred pregnant women were enrolled from the antenatal clinic and the ward of the Department of Obstetrics and Gynaecology. EOPE women (n=50) after clinical diagnosis as per American College of Obstetricians and Gynecologists (ACOG) guidelines [3] (inclusion criteria in Appendix) were enrolled as cases, and normotensive, non-proteinuric pregnant women (n=50) were enrolled as controls. Patients with chronic hypertension, chorioamnionitis, diabetes, renal disease, and cardiac disease were excluded from the study. Sample size was calculated using STATA 14 and GraphPad Prism Cloud. The protocol of the study was approved by the Institute Ethics Committee (Ref. No. IECPG-101/21.03.2018). The study duration was from 23.03.2018 to 24.07.2020. Written informed consent was obtained from all the enrolled women. Five ml of venous blood was collected from all 100 (50 each of patients and controls) recruited women (2.5 ml each in ethylenediamine tetraacetic acid (EDTA) and plain vial) followed by plasma and serum separation. Thirty cesarean-delivered placentae (15 each of patients and controls) were collected. Plasma samples and placentae were used to determine and compare the gene expression of MMPs 2, 9, TIMPs 1, 2, cystathionine beta-synthase, specificity protein-1, precursor miR-22 and miR-22-3p in the EOPE patients, and normotensive, non-proteinuric pregnant women by Real-Time Quantitative Reverse Transcription polymerase chain reaction (qRT-PCR). Protein levels of MMPs 2,9, TIMPs 1,2, CBS, and Sp1 in the serum samples were estimated by enzyme-linked immunosorbent assay (ELISA).

The protein expression of MMPs 2,9, TIMPs 1, 2, CBS, and Sp1 was observed by immunohistochemistry (IHC) and immunofluorescence (IF), and their levels were determined by western blot in the placentae of EOPE patients and normotensive, non-proteinuric controls. In all the collected placentae, the protein activity of MMP-2 and MMP-9 was assessed by gelatin gel zymography.

Real-time quantitative reverse transcription polymerase chain reaction

RNA extraction from plasma was carried out using TRIzol^TM^ reagent (Invitrogen®). Placental tissue (kept in RNA later) was blotted on absorbent paper, and 100 mg was washed in 1x phosphate-buffered saline (PBS) and taken for RNA isolation using Ambion, Invitrogen. cDNA was prepared using the RevertAid H-minus reverse transcriptase kit (Thermo Scientific). cDNA was amplified by quantitative RT-PCR (CFX96 Touch™ Real-Time PCR Detection System, BioRad). qRT-PCR reactions were carried out to determine mRNA expression of MMPs 2, 9, TIMPs 1, 2, CBS, Sp1, and gene expression of miR-22 (pre-miR-22 and miR-22-3p). Glyceraldehyde-3-phosphate dehydrogenase (GAPDH) mRNA and U6 small nuclear RNA were used as internal controls. Primers were designed by NCBI and confirmed by In-Silico PCR (Table 1).

**Table 1:** Primers designed by NCBI and confirmed by In-Silico PCR NCBI: National Centre for Biotechnology Information, PCR: Polymerase chain reaction, GAPDH: Glyceraldehyde 3-phosphate dehydrogenase.

Targets	Primers
MMP2	FP- CGTCTGTCCCAGGATGACATC RP- ATGTCAGGAGAGGCCCCATA
MMP9	FP- CGCCAGTCCACCCTTGT RP- CAGCTGCCTGTCGGTGAGA
TIMP1	FP- CCCTGGAACAGCCTGAGCTT RP- TGGATAAACAGGGAAACACTGTGC
TIMP2	FP- GCACATCACCCTCTGTGACTT RP- AGCGCGTGATCTTGCACT
CBS	FP- CTGAAGAACGAAATCCCCAA RP- GCCTCCTCATCGTTGCTCTT
Sp1	FP- GGAGAGCAAAACCAGCAGAC RP- AAGGTGATTGTTTGGGCTTG
GAPDH	FP- AGCCGAGCCACATC RP- TGAGGCTGTTGTCATACTTCTC
Precursor miR-22	FP-GGCTGAGCCGCAGTAGTTC RP-GCAGAGGGCAACAGTTCTTCAA
miR-22-3p	5’-AAGCUGCCAGUUGAAGAACUGU-3’
U6	FP-CTCGCTTCGGCAGCACA RP-AACGCTTCACGAATTTGCGT

ELISA

The levels of MMPs 2, 9, TIMPs 1, 2, CBS, and Sp1 were estimated in the serum of EOPE and normotensive pregnant women by sandwich ELISA [MMPs 2,9, TIMPs 1,2 (R & D Systems), CBS, Sp1 (G-Biosciences)].

Immunohistochemistry

Paraffin tissue blocks were sectioned on a microtome (Thermo ScientificTM HM 325) (5µm sections) and were taken on Poly-L-lysine (Sigma) coated slides. UltraVision™ Quanto Detection System HRP DAB (Thermo, TL-125-QHD) was used to determine protein expression of MMPs 2,9, TIMPs 1,2, CBS, and Sp1. Primary antibodies to MMP-2 (Abcam) at a dilution of 1:500, MMP-9 (Abcam) at a dilution of 1:500, TIMP-1 (Thermo) at a dilution of 1:100, TIMP-2 (Novus Biologicals) at a dilution of 1:100, CBS (Abcam) at a dilution of 1:400 and Sp1 (Merck) at a dilution of 1:250 were used. Slides were counterstained with hematoxylin, dehydrated in graded ethanol, cleared, and coverslips were applied. Mounting was done using dibutylphthalate polystyrene Xylene (DPX). Stained slides were observed under Nikon Eclipse Ti-S elements microscope using NiS-AR software (version 5.1).

Immunofluorescence

Paraffin tissue blocks were sectioned on a microtome (Thermo ScientificTM HM 325; Thermo Scientific, MA, USA) and were taken on Poly-L-lysine (Sigma) coated slides. Two changes of xylene (each for five minutes) followed by two changes of absolute alcohol (each for three minutes) and subsequently one change of 90% alcohol (for one minute) were given. Slides were rinsed with distilled water. Antigen retrieval was done with sodium citrate buffer (15 minutes at 95-100°C) followed by treatment with tris-buffered saline (TBS)Tx (two minutes) (two times). BSA (blocking agent) was applied on slides for 35 minutes followed by overnight incubation with primary antibodies (MMP-2 (Abcam, Cambridge, UK) at a dilution of 1:100, MMP-9 (Abcam) at a dilution of 1:100, TIMP-1 (Thermo Scietific) at a dilution of 1:10, TIMP-2 (Novus Biologicals, CO, USA) at a dilution of 1:25, CBS (Abcam) at a dilution of 1:200 and Sp1 (Merck, Darmstadt, Germany) at a dilution of 1:50] at 4°C. Slides were then rinsed with phosphate-buffered saline with Tween 20 (PBST) followed by incubation with secondary antibodies (MMP-2, MMP-9, TIMP-2, CBS, Sp1 (fluorescein Isothiocyanate (FITC) conjugated, Abcam), TIMP-1 (tetramethylrhodamine isothiocyanate (TRITC) conjugated, Abcam) for 2.5 hours and then washed with PBS. Mounting was done by fluoroshield mounting media with 4′,6-diamidino-2-phenylindole (DAPI (Abcam). Stained slides were observed under Nikon Eclipse Ti-S elements microscope using NiS-AR software (version 5.1).

Western blot

Protein extraction (from the placental tissues of both cases and controls) was done with RIPA buffer (Thermo), adding the protease inhibitor cocktail. Separating and stacking gels were prepared, 3x non-reducing sample buffer was added to the samples, followed by denaturation at 95˚C for 5 minutes, and the samples were loaded along with protein molecular marker to the wells. Subsequently, running the gel at 50 V (vertical electrophoresis apparatus, BioRad) in electrophoresis buffer for 3-3.5 hours until good band separation is achieved. Nitrocellulose membrane was used for the transfer of gel products onto the membrane in transfer buffer for 90 minutes. Membrane was washed with TBST, followed by blocking in 5% bovine albumin serum (BSA) TBST for 90 minutes. Overnight incubation was done with primary antibodies (MMP-2 (Abcam) at a dilution of 1:1000, MMP-9 (Abcam) at a dilution of 1:1000, TIMP-1 (Thermo) at a dilution of 1:200, TIMP-2 (Novus Biologicals) at a dilution of 1:200, CBS (Abcam) at a dilution of 1:1000 and Sp1 (Merck) at a dilution of 1:400] at 4°C. Washing was done with TBST, followed by incubation with secondary antibodies (Abcam) for three hours, and then washed with TBST. Enhanced chemiluminescence (ECL) kit (Thermo) was used for visualization of bands on the Densitometer (Protein Simple).

Gelatin gel zymography

Protein was extracted from placentae using radio-immunoprecipitation assay (RIPA) buffer (Thermo), adding the protease inhibitor cocktail, and 7.5% acrylamide gel was prepared containing gelatin, then 5x non-reducing sample buffer was added to each of the isolated protein samples from patients and controls. A 10 μl protein sample was loaded into each well. Protein molecular weight marker (Thermo) was loaded, subsequently, running the gel at 90 V (vertical electrophoresis apparatus, BioRad) in electrophoresis buffer until good band separation was achieved. Gel was washed (2 x 30 minutes) with washing buffer, then rinsed in incubation buffer for 5-10 minutes at 37°C with agitation. Fresh incubation buffer was added to the gel, followed by incubation at 37°C for 24 h. Gel was stained with Coomassie blue for 30 minutes and rinsed with water. Incubation was done with a destaining solution to visualise the bands showing pro and active forms of MMP-2 and MMP-9 in the study groups.

Statistical analysis

Data were analyzed using STATA 14 (Stata Statistical Software, College Station, TX) and GraphPad Prism 8 (www.graphpad.com). Relative quantification cycles of the gene of interest (ΔCq) were calculated by ΔCq = Cq (target) - Cq (reference). Relative mRNA expression with respect to the internal control gene was calculated by 2^-ΔCq^. Paired t-test and Wilcoxon matched-pairs signed rank test were used to compare the average level of the variable between two groups; a p-value < 0.05 was considered statistically significant.

## Results

Clinical characteristics of EOPE participants and normotensive, non-proteinuric controls are mentioned in Table 2.

**Table 2:** Maternal study population-clinical characteristics n= number of subjects, data presented as mean±SD, p values between groups were evaluated by Paired t-test, p<0.05 considered statistically significant, *p≤0.05, **p≤0.01, ***p≤0.001, ****p≤0.0001, ns: not significant. EOPE: Early-onset preeclampsia.

Clinical characteristics	EOPE (n=50)	Normotensive, non-proteinuric controls (n=50)	p value
Maternal age (years)	28.38 ± 4.29	28.38 ± 4.66	1.00 (ns)
Gestational age (onset) (weeks)	31.30 ± 2.46	31.53 ± 2.81	0.48 (ns)
Gestational Age (delivery) (weeks)	34.26 ± 4.20	37.57 ± 1.26	0.002**
Systolic blood pressure (mmHg)	146.82 ± 10.59	115.26 ± 7.97	p<0.0001****
Diastolic blood pressure (mmHg)	95.8 ± 8.97	74.72 ± 7.51	p<0.0001****
Protein (dipstick)	27 EOPE patients- 1+, 13 EOPE patients- 2+, 10 EOPE patients- 3+	Nil or traces	Not applicable
Placental weight (grams)	440 ± 47.71	507.8 ± 9.60	p=0.04*

The gestational age at delivery was 34.26 ± 4.20 weeks in cases compared to 37.57 ± 1.26 weeks in controls, showing a significant difference. Placental weight captured post-delivery was 440 ± 47.7 in the EOPE participants compared with 507.8 ± 9.60 in normotensive controls, indicating a significant difference between the two study arms.

MMPs 2, 9 and TIMPs 1, 2 levels in the circulating blood of EOPE

**Figure 1:**
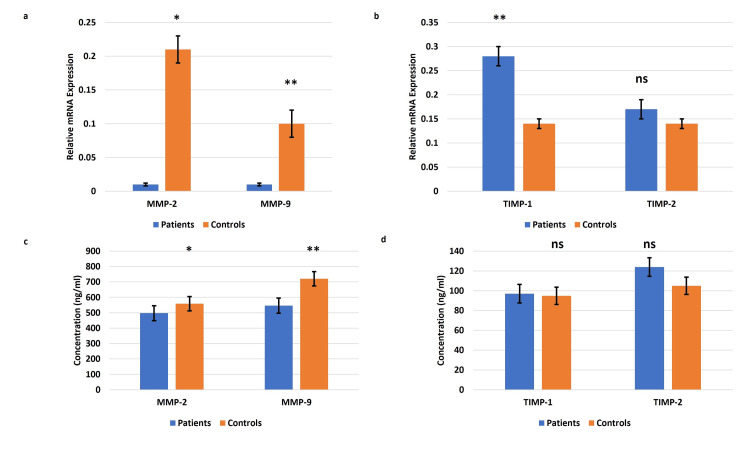
Gene expression details in the blood samples of the EOPE cases Gene expression of matrix metalloproteinases (MMPs) 2, 9 (a) and tissue inhibitors of metalloproteinase (TIMPs) 1, 2 (b) in the blood samples of the early-onset preeclampsia (EOPE) cases. Bar diagrams represent the relative mRNA expression of MMPs 2, 9 (a) and TIMPs 1, 2 (b) in plasma samples of EOPE and normotensive, non-proteinuric controls. Glyceraldehyde-3-phosphate dehydrogenase (GAPDH) was used as a positive control. The protein expression of MMPs 2, 9 (c) and TIMPs 1, 2 (d) in the blood samples of the EOPE. Bar diagrams represent serum levels of MMPs 2, 9 (c) and TIMPs 1,2 (d) proteins in serum samples of EOPE cases and normotensive, non-proteinuric controls. Data presented as mean ± SEM. Wilcoxon matched-pairs signed rank test was applied, *p≤0.05 was considered statistically significant, *p≤0.05, **p≤0.01, ***p≤0.001, ****p≤0.0001, ns: not significant.

The qRT-PCR data revealed a significant reduction of mRNA expression of the MMPs 2, 9 in the plasma samples from pregnancies complicated by EOPE as compared to their normotensive, non-proteinuric controls. Increased trends in the expression of TIMPs 1, 2 were observed in the EOPE cases. Corroborating the levels of the transcripts, circulating protein levels of MMPs 2, 9 were found to be reduced notably in EOPE with higher trends of TIMPs 1 and 2 compared to controls (Figure 1).

Placental expression of MMPs 2, 9 and TIMPs 1, 2 in EOPE

**Figure 2:**
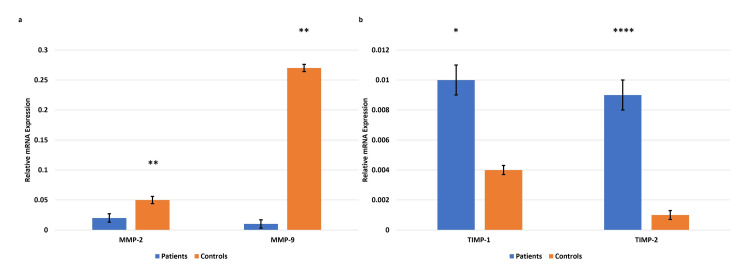
mRNA expression in the placental tissue samples from pregnancies complicated by EOPE mRNA expression of matrix metalloproteinases (MMPs) 2, 9 (a) and tissue inhibitors of metalloproteinase (TIMPs) 1, 2 (b) in the placental tissue samples from pregnancies complicated by early-onset preeclampsia (EOPE). Bar diagrams represent the relative mRNA expression of MMPs 2, 9 (a) and TIMPs 1,2 (b) in placentae from EOPE and normotensive, non-proteinuric controls. Glyceraldehyde-3-phosphate dehydrogenase (GAPDH) was used as a positive control. Data presented as mean ± SEM. Wilcoxon matched-pairs signed rank (MMPs 2, 9, TIMP-1) and paired t (TIMP-2) tests were applied, *p≤0.05 was considered statistically significant, *p≤0.05, **p≤0.01, ***p≤0.001, ****p≤0.0001, ns: not significant.

The qRT-PCR data revealed a significant reduction of the mRNA expression of MMPs 2, 9 in the placental tissue samples from pregnancies complicated by EOPE as compared to the normotensive, non-proteinuric controls. Contrary to the transcript levels of MMPs 2 and 9, the levels of their inhibitors, TIMPs 1 and 2 were found to be increased in EOPE compared to controls (Figure 2).

**Figure 3:**
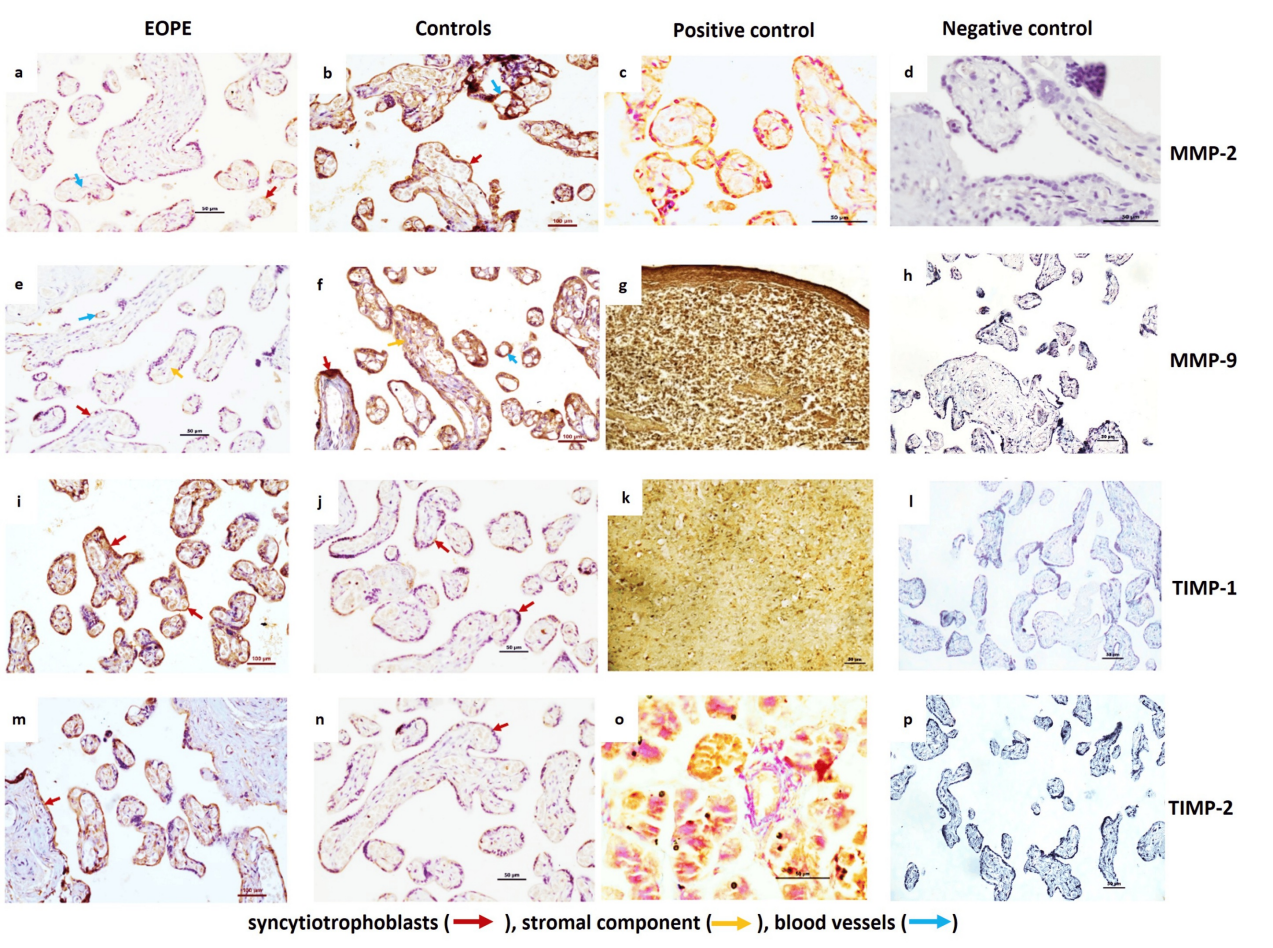
Protein expression in the samples Protein expression of matrix metalloproteinases (MMPs) 2, 9, and their feedback inhibitor tissue inhibitors of metalloproteinase (TIMPs) 1, 2  as analysed by immunohistochemistry (IHC). Representative IHC images of placentae from early-onset preeclampsia (EOPE) (a, e, i, m) and normotensive, non-proteinuric controls (b, f, j, n) showing MMP-2, MMP-9, TIMP-1 and TIMP-2 localization in syncytiotrophoblasts, stromal component, and blood vessels. Positive controls for MMP-2 (human placenta (c)), MMP-9 (human spleen (g)), TIMP-1 (rat brain (k)) and TIMP-2 (human pancreas (o)). Negative controls for MMP-2 (d), MMP-9 (h), TIMP-1 (l) and TIMP-2 (p), Scale Bar: 50 µm.

**Figure 4:**
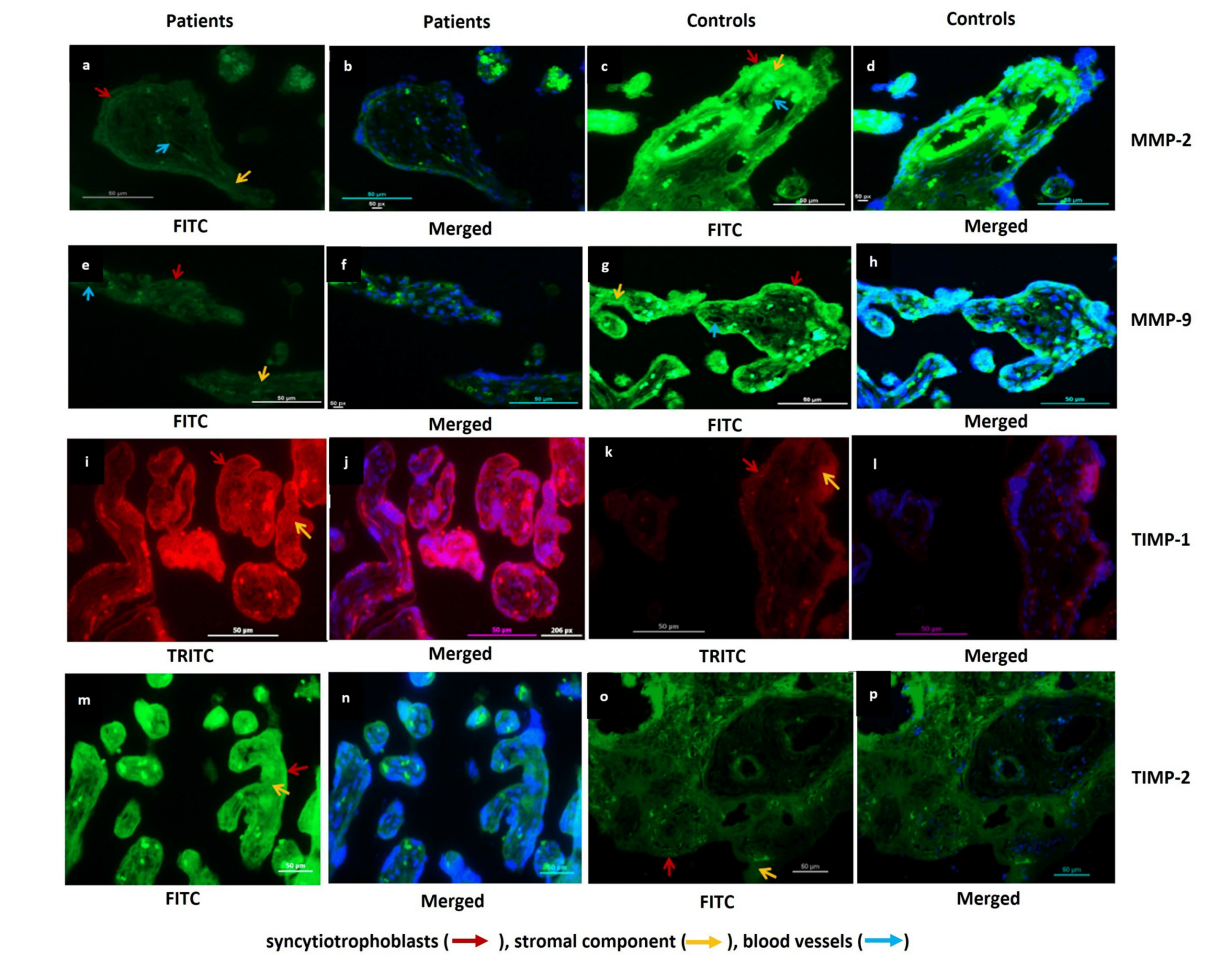
Protein expressions in samples analysed by IF Protein expression of matrix metalloproteinases (MMPs) 2, 9  and tissue inhibitors of metalloproteinase (TIMPs) 1, 2 as analysed by immunofluorescence (IF) in EOPE and normotensive controls. Representative IF images of placentae from EOPE and normotensive, non-proteinuric controls showing MMP-2 (a, c (FITC stained), b, d (merged)], MMP-9 (e, g (FITC stained), f, h (merged)), TIMP-1 (i, k (TRITC stained), j, l (merged)) and TIMP-2 (m, o (FITC stained), n, p (merged)) localization in syncytiotrophoblasts, stromal component and blood vessels. Nuclei were stained by 4′,6-diamidino-2-phenylindole (DAPI); Scale Bar: 50 µm, FITC: Fluorescein isothiocyanate, TRITC: Tetramethylrhodamine isothiocyanate.

Similarly, protein expression of MMPs 2,9 as analysed by immunohistochemistry (IHC) and immunofluorescence (IF), also reduced notably in EOPE patients with an increase in the protein expression of TIMPs 1, 2 compared to controls. Immunostaining demonstrated weaker expression of MMP-2 and MMP-9 in syncytiotrophoblasts, stroma, and around blood vessels in the placentae from EOPE compared to controls. TIMPs 1 and 2 were found strongly localized in the stromal component and syncytium in the placentae from EOPE compared to control placentae (Figures 3, 4).

**Figure 5:**
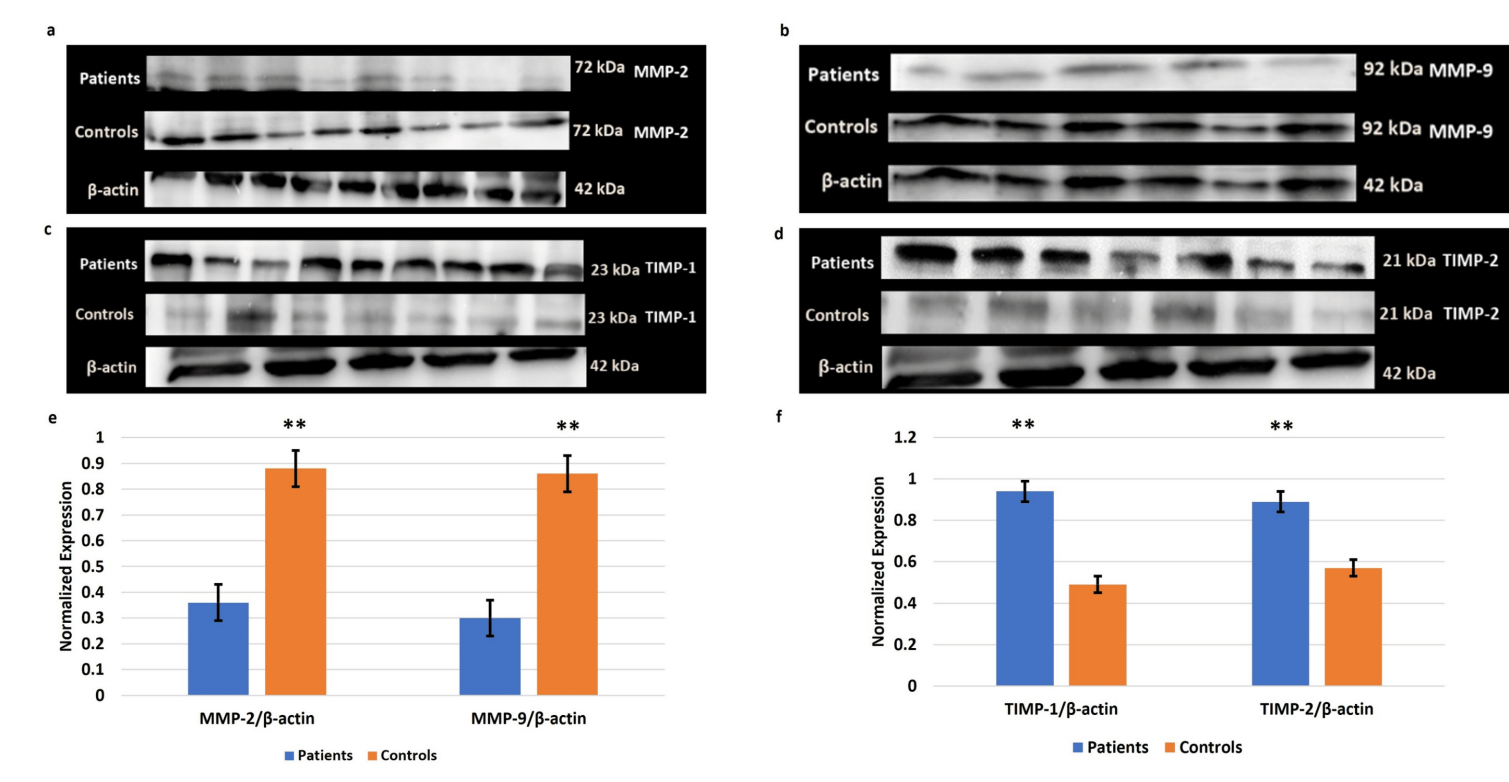
Immunoblot study of the samples Immunoblot of matrix metalloproteinases (MMPs) 2, 9 (a, b) and tissue inhibitors of metalloproteinase (TIMPs) 1, 2 (c, d) in the placentae from early-onset preeclampsia (EOPE) as compared to normotensive, non-proteinuric control placentae. Representative images of immunoblot showing the protein expression of MMP-2 (a), MMP-9 (b), TIMP-1 (c) and TIMP-2 (d) in placental tissues of EOPE and normotensive, non-proteinuric controls. Bar diagrams represent the normalized values of MMPs 2, 9 (e) and TIMPs 1, 2 (f) with respect to β-actin (loading control). Data presented as mean ± SEM. Statistical analysis was done using the Wilcoxon matched-pairs signed rank (MMPs 2, 9, TIMP-1) and paired t (TIMP-2) tests; *p≤0.05 was considered statistically significant. *p≤0.05, **p≤0.01, ***p≤0.001, ****p≤0.0001, ns: not significant

Immunoblot of MMPs 2 and 9 showed significant downregulation in EOPE, with upregulation of TIMPs 1, 2 in the placentae from EOPE as compared to normotensive, non-proteinuric control placentae (Figure 5).

**Figure 6:**
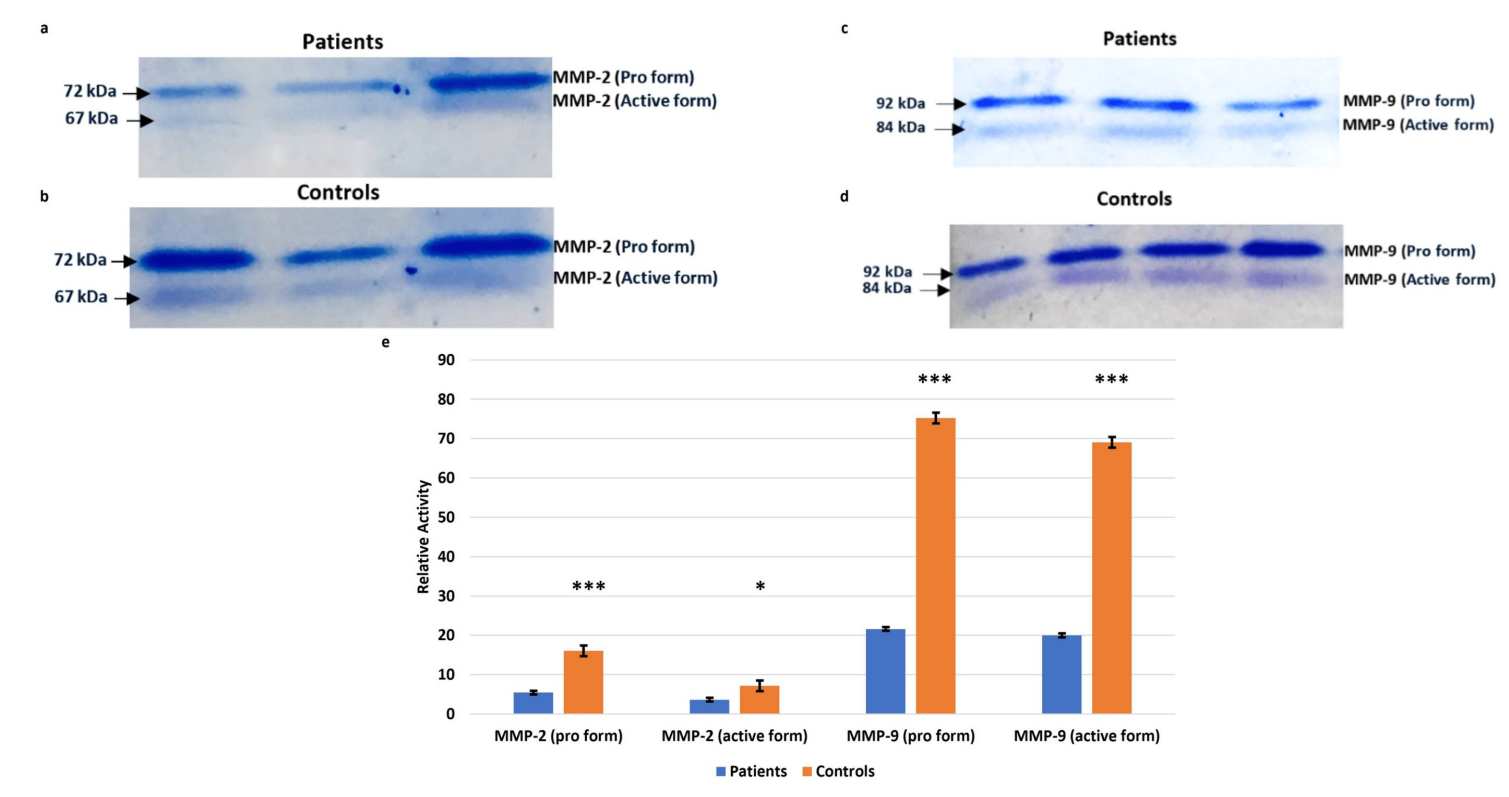
MMP-2 and MMP-9 activity in placentae from EOPE Matrix metalloproteinases (MMP-2) (a, b) and MMP-9 (c, d) activity in placentae from early-onset preeclampsia (EOPE) as compared to placentae from controls. Representative zymograms depicting gelatinase activity on the maternal side of placentae from EOPE and normotensive, non-proteinuric controls. Comparison of pro and active forms of MMP-2 and MMP-9 between patients and controls was done by the Wilcoxon matched-pairs signed rank test (e). Data expressed in the form of mean ± SEM, *p≤0.05 was considered statistically significant, *p≤0.05, **p≤0.01, ***p≤0.001, ****p≤0.0001, ns: not significant.

MMPs 2, 9 activity (pro and active forms) assayed by gelatin gel zymography was significantly lower in placentae from EOPE than in controls, corroborating the immunofluorescence and immunoblot results (Figure 6).

Expression levels of cystathionine β-synthase (CBS) and specificity protein 1 (Sp1) in the blood samples and placentae of EOPE

**Figure 7:**
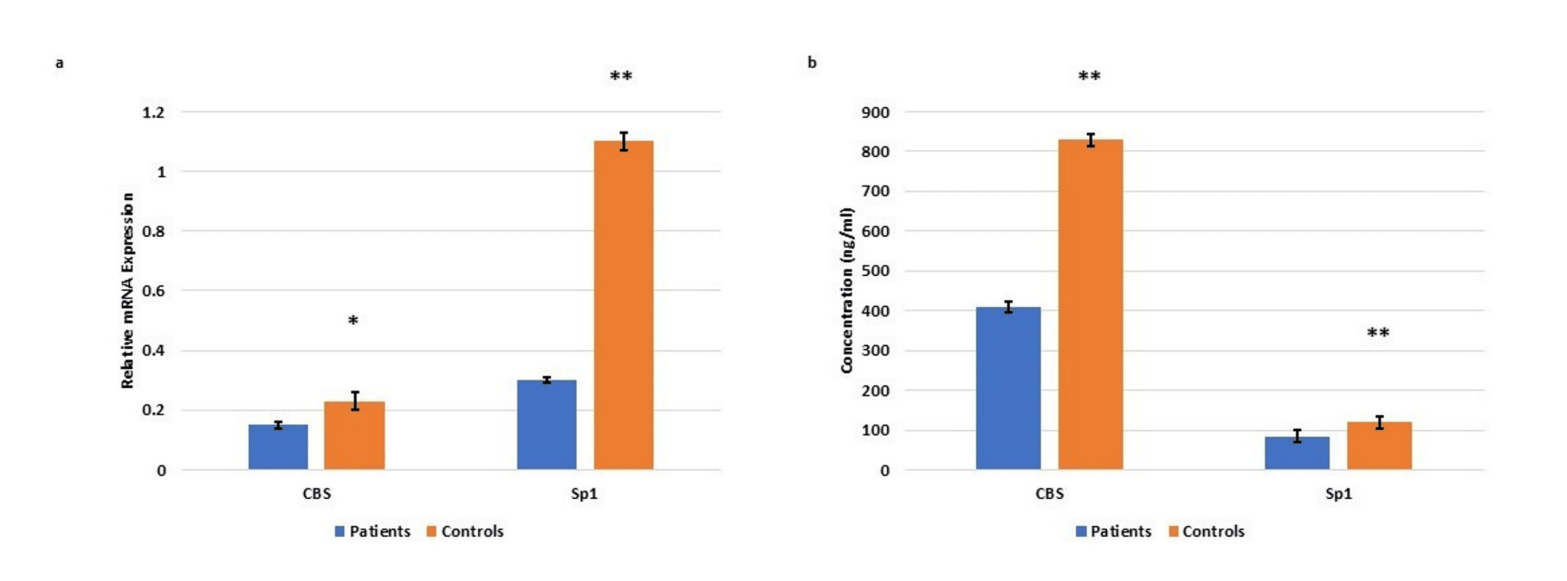
Gene expression of CBS and Sp1 in the EOPE patient samples Gene expression of Cystathionine β-synthase (CBS) and Specificity protein 1 (Sp1) in the circulating blood of the early-onset preeclampsia (EOPE) patients (a). Bar diagrams represent the relative mRNA expression of CBS and Sp1 in plasma samples of EOPE and normotensive, non-proteinuric controls (a). GAPDH was used as a positive control. Protein levels of CBS and Sp1  in the circulating blood of EOPE patients (b). Bar diagrams represent the protein concentration of CBS and Sp1 in serum samples of early-onset preeclamptic patients and normotensive, non-proteinuric controls (b). Data presented as mean ± SEM. Wilcoxon matched-pairs signed rank test was applied, *p≤0.05 was considered statistically significant, *p≤0.05, **p≤0.01, ***p≤0.001, ****p≤0.0001, ns: not significant.

The qRT-PCR data revealed a significant reduction in the mRNA expression of CBS and Sp1 in plasma samples from EOPE compared to the normotensive, non-proteinuric controls. Similarly, circulating protein levels of CBS and Sp1 assayed using ELISA were significantly reduced in EOPE compared to controls (Figure 7).

**Figure 8:**
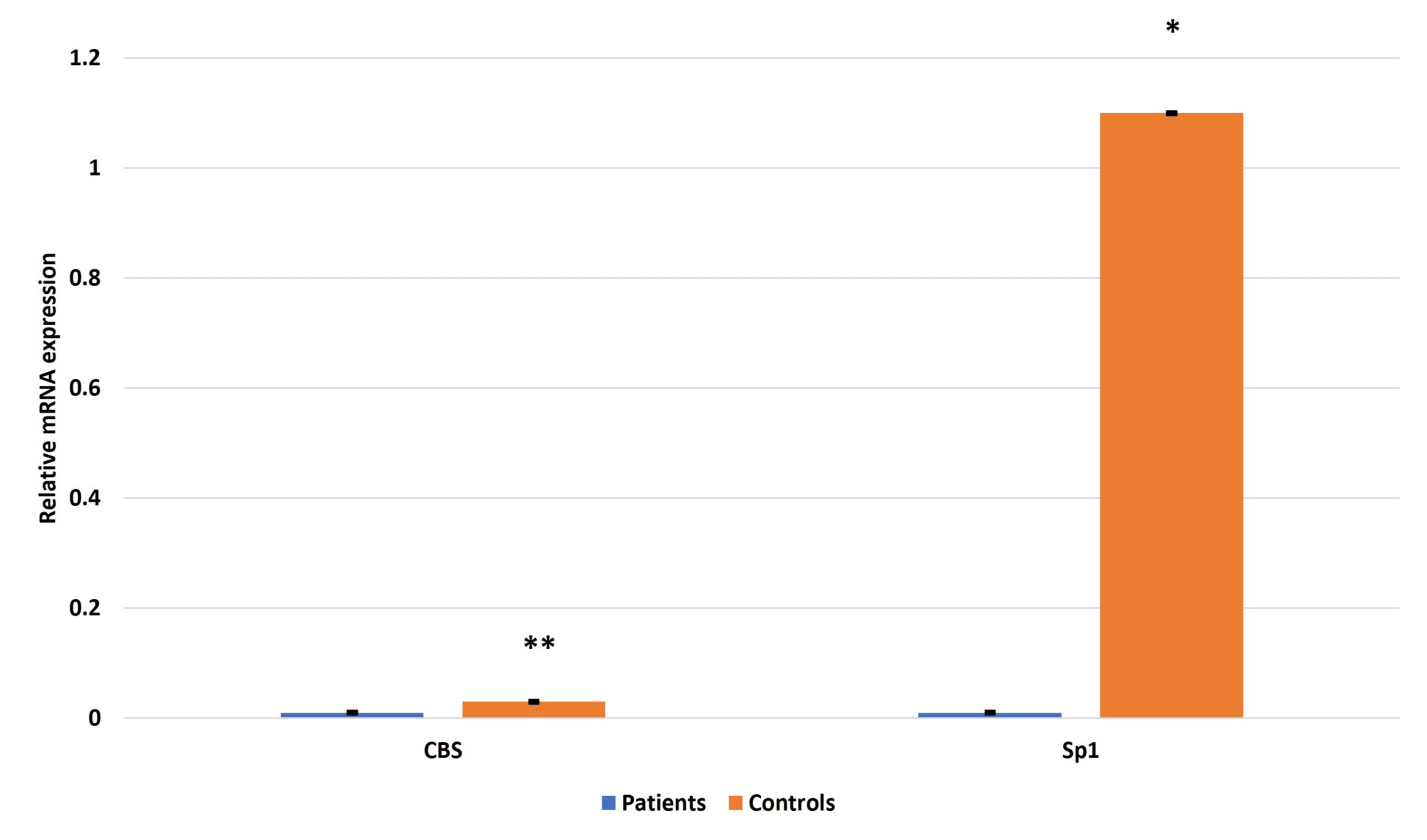
mRNA expression of CBS and Sp1 in placental tissue samples mRNA expression of cystathionine β-synthase (CBS) and specificity protein 1 (Sp1) in placental tissue samples from pregnancies complicated by early-onset preeclampsia (EOPE) as compared to the normotensive, non-proteinuric controls. Bar diagrams represent the relative mRNA expression of CBS and Sp1 in placentae of EOPE patients and normotensive, non-proteinuric controls.  GAPDH was used as a positive control. Data presented as mean ± SEM. Wilcoxon matched-pairs signed rank test was applied, *p≤0.05 was considered statistically significant, *p≤0.05, **p≤0.01, ***p≤0.001, ****p≤0.0001, ns: not significant.

Transcript profiles of CBS and Sp1 in the placentae of EOPE revealed significantly lower mRNA expression compared to the normotensive, non-proteinuric controls [Figure 8].

**Figure 9:**
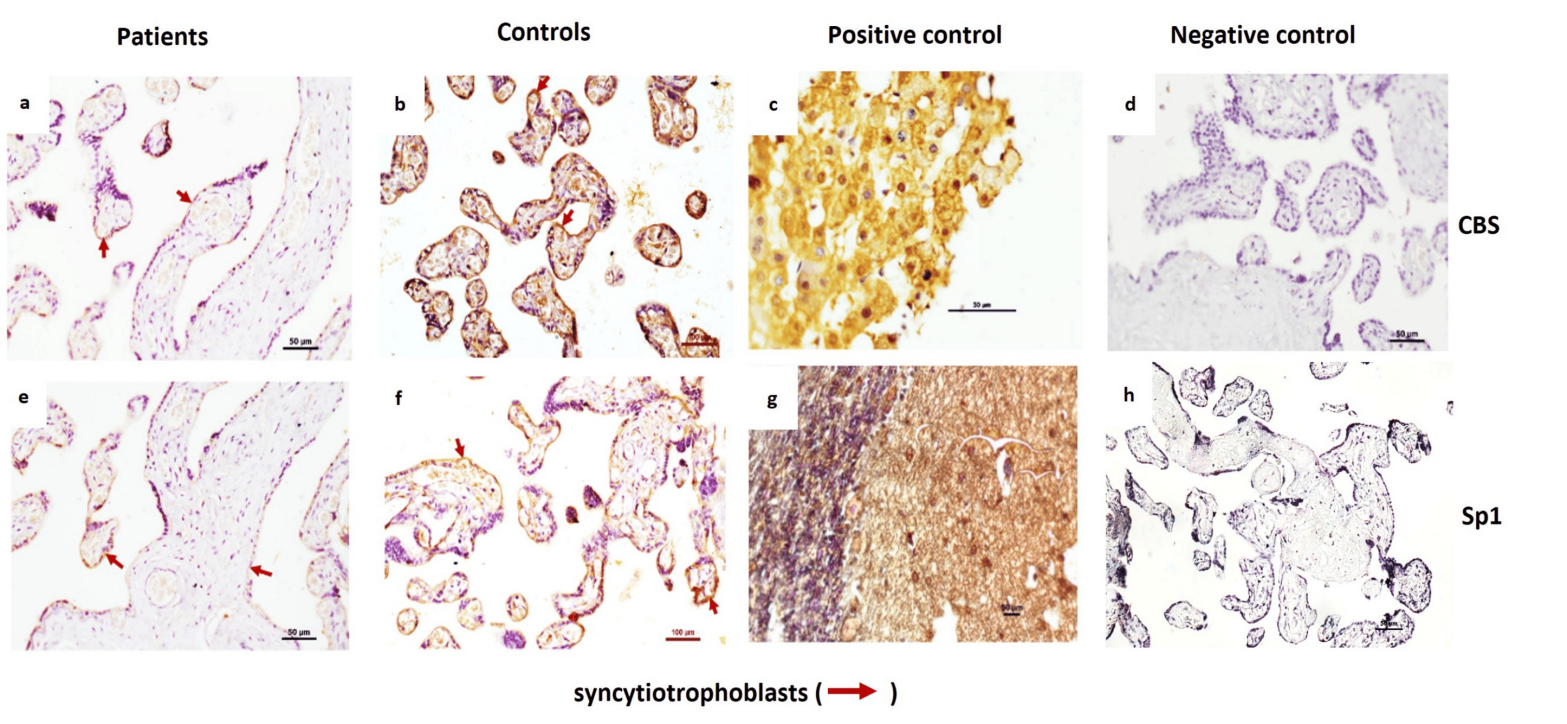
IHC of CBS and Sp1 in EOPE patient samples Immunohistochemistry (IHC) of cystathionine β-synthase (CBS) and specificity protein 1 (Sp1) in early-onset preeclampsia (EOPE) patients and normotensive control placentae. Representative IHC images of placentae from EOPE patients (a, e) and normotensive, non-proteinuric controls (b, f) showing CBS and Sp1 localization in syncytiotrophoblasts. Positive controls for CBS (human liver (c)) and Sp1 (human cerebellum (g)). Negative controls for CBS (d) and Sp1 (h), Scale Bar: 50 µm.

**Figure 10:**
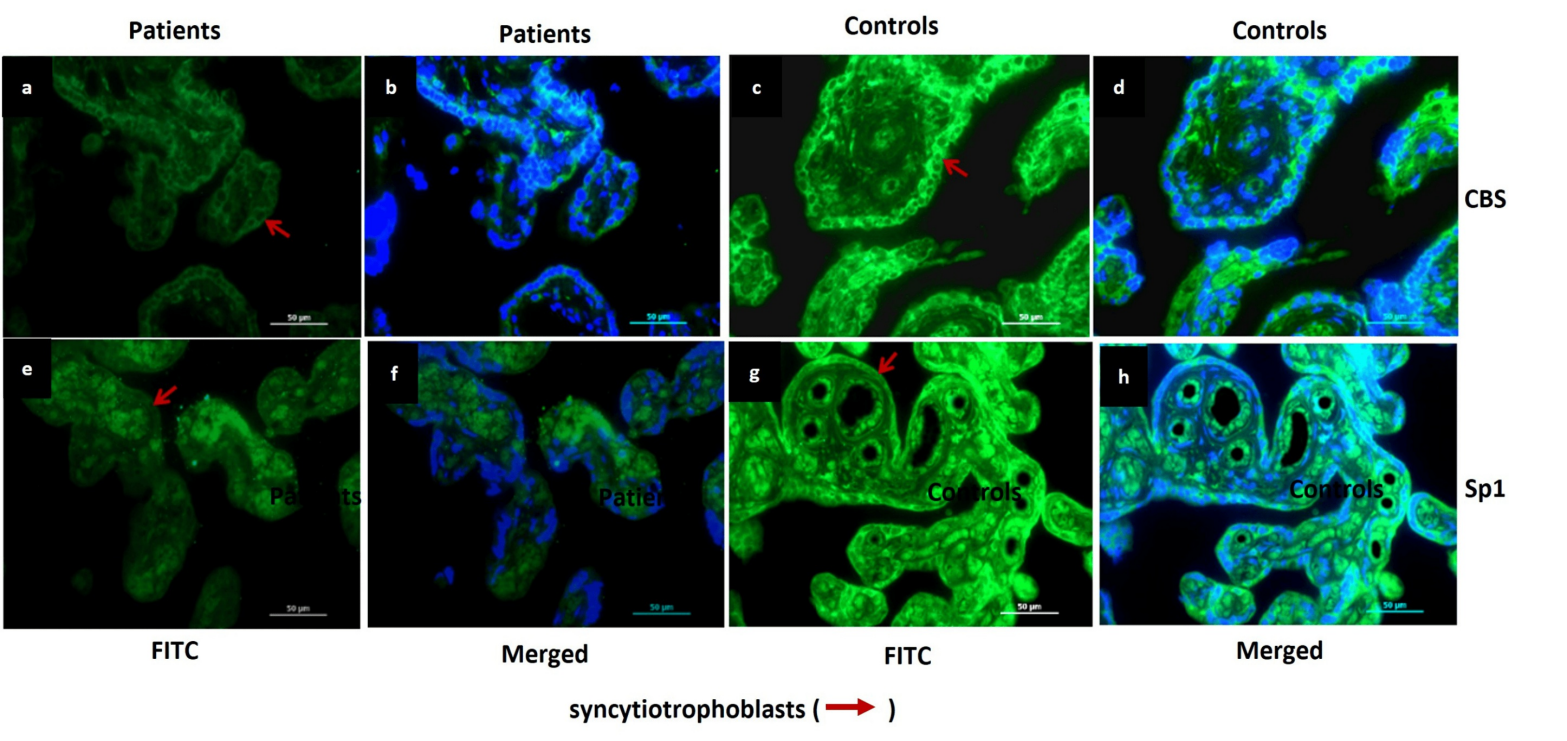
Protein expression of CBS and Sp1 analysed by IF in EOPE patient samples Protein expression of cystathionine β-synthase (CBS) and specificity protein (Sp1), as analysed by immunofluorescence (IF) was significantly reduced in early-onset preeclampsia (EOPE) patients in comparison to controls. Representative IF images of placentae from early-onset preeclamptic patients and normotensive, non-proteinuric controls showing CBS (a, c (fluorescein Isothiocyanate (FITC) stained), b, d (merged)) and Sp1 (e, g (FITC stained), f, h (merged)) localization in syncytiotrophoblasts. Nuclei were stained by 4′,6-diamidino-2-phenylindole (DAPI); Scale Bar: 50 µm.

Compared to control placentae, IHC and IF staining demonstrated weaker expression of CBS and Sp1 in the syncytiotrophoblasts in the placentae of EOPE (Figures 9, 10).

**Figure 11:**
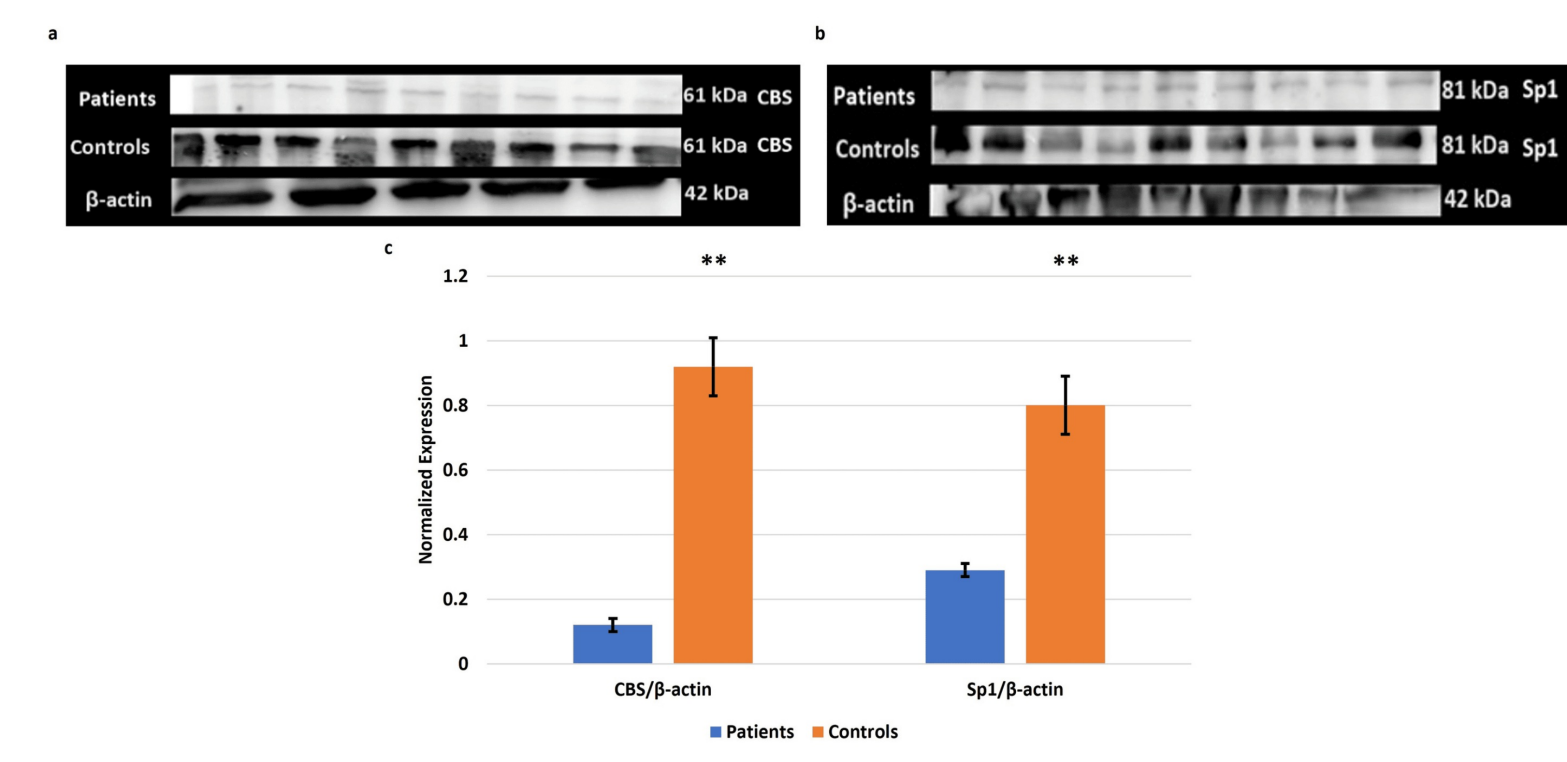
Immunoblot of CBS (a) and Sp1 (b) in the placentae from EOPE patients Immunoblot of cystathionine β-synthase (CBS) (a) and specificity protein 1 (Sp1) (b) in the placentae from early-onset preeclampsia EOPE and normotensive, non-proteinuric control placentae. Representative images of immunoblot showing the protein expression of  CBS (a) and Sp1 (b) in placental tissues of EOPE patients and normotensive, non-proteinuric controls. The bar diagram represents the normalized values of CBS and Sp1 with respect to β-actin (c). Data presented as mean ± SEM. Statistical analysis was done using the Wilcoxon matched-pairs signed rank test, *p≤0.05 was considered statistically significant, *p≤0.05, **p≤0.01, ***p≤0.001, ****p≤0.0001, ns: not significant.

The immunoblot showed a similar trend of significant downregulation in the expression of CBS and Sp1 in the placentae from EOPE compared to normotensive, non-proteinuric control placentae (Figure 11).

Gene expression of precursor miR-22 and miR-22-3p in circulating plasma and placentae associated with EOPE

**Figure 12:**
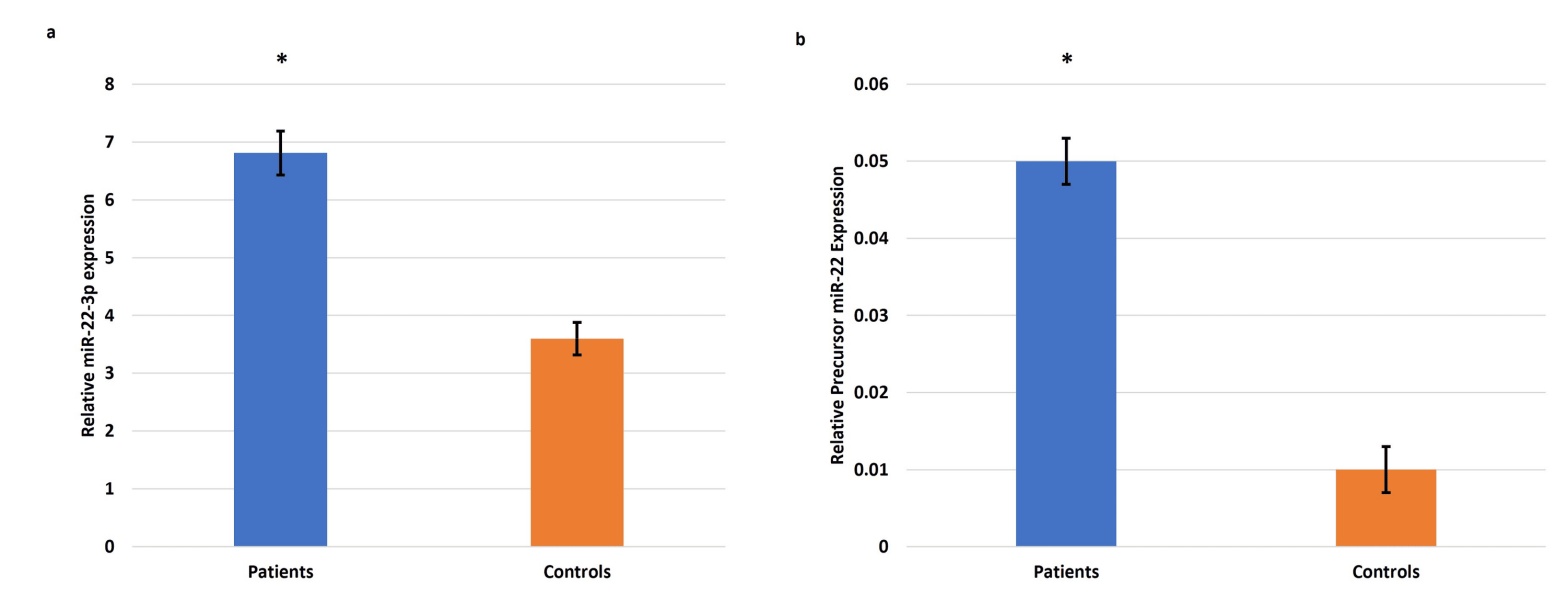
Gene expression of miR-22-3p and precursor miR-22 in the circulating blood of EOPE patients and controls Gene expression of microRNA-22 (miR-22)-3p (a) and precursor miR-22 (b) in the circulating blood of early-onset preeclampsia (EOPE) patients and controls. Bar diagrams represent the relative gene (miR-22-3p (a), precursor miR-22 (b)) expression. U6 was used as a positive control. Data presented as mean ± SEM. Wilcoxon matched-pairs signed rank test was applied, *p≤0.05 was considered statistically significant, *p≤0.05, **p≤0.01, ***p≤0.001, ****p≤0.0001, ns: not significant.

**Figure 13:**
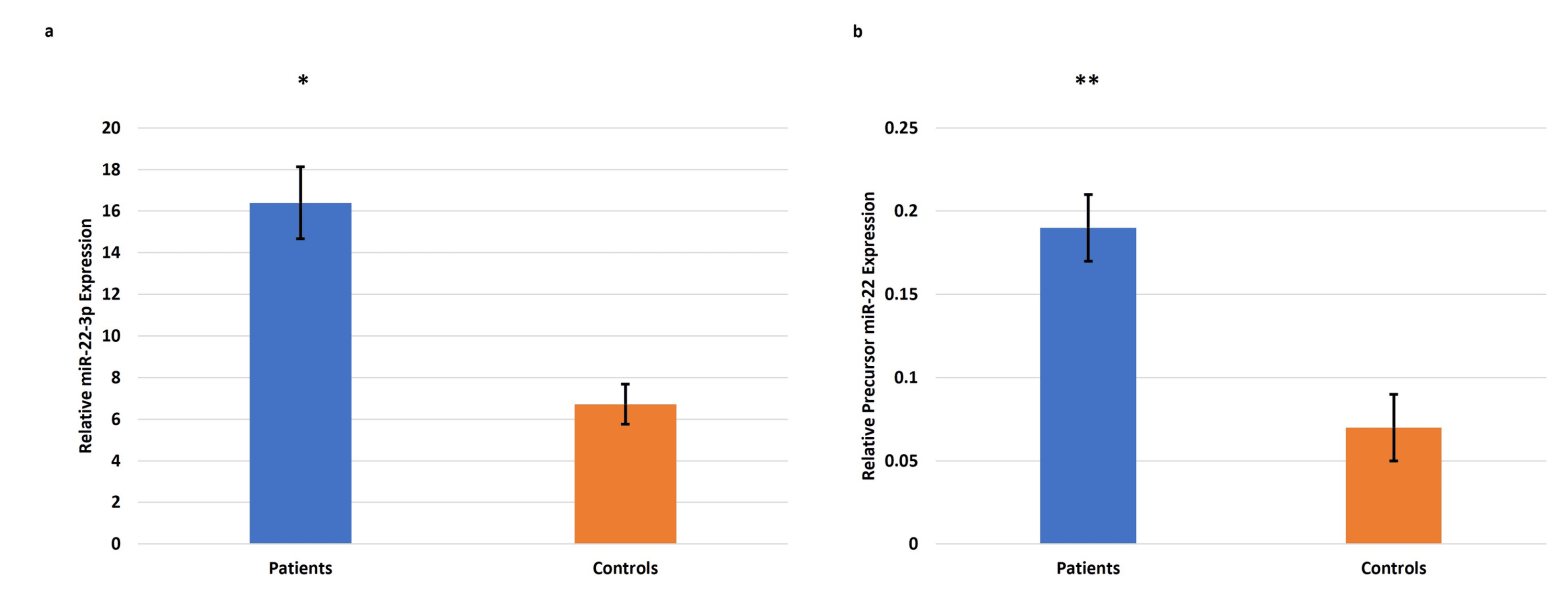
Gene expression of miR-22-3p and precursor miR-22 in the placentae of EOPE patients Gene expression of microRNA-22 (miR-22)-3p (a) and precursor miR-22 (b) in the placentae of early-onset preeclampsia (EOPE) patients and controls. Bar diagrams represent the relative gene (miR-22-3p (a), precursor miR-22 (b)) expression. U6 was used as a positive control. Data presented as mean ± SEM. Wilcoxon matched-pairs signed rank test was applied, *p≤0.05 was considered statistically significant, *p≤0.05, **p≤0.01, ***p≤0.001, ****p≤0.0001, ns: not significant.

The transcript profile of miR-22 (precursor miR-22 and miR-22-3p) expression using qRT-PCR revealed significant upregulation in the plasma samples from pregnancies complicated by EOPE compared to the normotensive, non-proteinuric controls (Figure 12). A similar upregulated trend of miR-22 (precursor miR-22 and miR-22-3p) was observed in placentae from pregnancies complicated by EOPE as compared to the normotensive, non-proteinuric control placentae (Figure 13).

## Discussion

Preeclampsia (PE), one of the most common pregnancy complications, is classified into early-onset (EOPE) and late-onset (LOPE), which have different aetiologies and are regarded as different forms of disease [13]. EOPE is the most severe clinical variant of the disease, occurring in 5-20% of all cases of PE. It is associated with impaired fetal growth, fetal pathology, uterine blood circulation, the small size of the placenta, preterm delivery, neonatal morbidity, and mortality [14]. EOPE is associated with impaired trophoblast invasion, immune maladaptation, and increased markers of endothelial dysfunction [14]. 

The current study reports significantly reduced mRNA and protein expression/levels of MMPs 2 and 9 in EOPE participants as compared with controls. Elevated levels of TIMP-1 and TIMP-2 in the same study population corroborate these findings. A previous study using a comparatively smaller sample size (30 each of EOPE patients and controls) from our group found that early-onset preeclamptic patients had down-regulated MMP-9 and up-regulated TIMP-1 expression in comparison to controls [15]. Reduced levels of CBS and Sp1 in the EOPE patients further establish the requirement of H₂S for the regulation of MMPs. The reduced levels of Sp1 associated with significantly upregulated expression of miR-22 in the EOPE participants in comparison to controls further suggest that the MMPs 2, 9, TIMPs 1, 2, CBS, Sp1, and miRNA-22 axis may be the underlying etiology of EOPE.

PE is characterized by disturbed and inadequate remodeling of the maternal spiral arteries by inadequate invasion of the trophoblast cells, thus reducing blood flow to the intervillous space, leading to systemic hypertension and fetal hypoxia [16]. To facilitate trophoblast cell invasion and adequate spiral artery remodeling, biochemical mediators such as matrix metalloproteinases (MMPs) are essential [17], which belong to a family of 17 zinc-dependent endopeptidases [18]. Specifically, MMP-2 and MMP-9 are involved in the remodeling of placental and uterine arteries, with abnormal expression of these MMPs observed in hypertensive disorders of pregnancy [19,20]. In the present study, we observed significantly downregulated mRNA and protein expression/levels of MMPs 2, 9 in the circulating blood and placentae of EOPE patients compared to normotensive, non-proteinuric controls. Reduced levels of MMP-9 protein have been reported in PE placental tissues collected at delivery compared to placentae from controls [21]. In addition, umbilical cord tissues have been reported to show lower MMP-2 and MMP-9 levels in PE compared to healthy controls ^[22]^.

MMP activity is regulated at the level of transcription, activation of latent forms, and inhibition by endogenous MMP inhibitors (tissue inhibitors of metalloproteinases; TIMPs) [23]. MMPs 2, 9 activity is regulated at different levels by interaction with TIMPs 2,1 [19,24]. In the present study, we observed significantly elevated mRNA and protein expression/levels of TIMPs 1 and 2 in the circulating blood collected at diagnosis and also in the placentae from EOPE patients compared to normotensive, non-proteinuric controls. Plasma levels of TIMP-1 have been reported to be up-regulated in PE patients compared to normotensive pregnant women [25,26]. Protein concentration of TIMP-2 was found to rise in the plasma and amniotic fluid of preeclamptic patients compared to healthy pregnant women [27,28].

The molecular mechanism behind the aberrant expression of MMPs 2, 9 and their inhibitors (TIMPs 1, 2) in pregnancies complicated by EOPE is unexplored. Liu et al. 2017 studied the effects of exogenous sodium hydrogen sulfide (NaHS) on the proliferation and invasion of human bladder cancer cells [29]. Treatment of human bladder cancer cells with different concentrations of exogenous NaHS (H₂S donor) promoted cell proliferation and invasion [29]. The study suggested that exogenous H₂S could promote cell proliferation and invasion by upregulating the expression of MMP-2 and MMP-9 in human bladder cancer EJ cells [29]. Cystathionine β-synthase (CBS) regulates homocysteine metabolism and contributes to hydrogen sulfide (H₂S) biosynthesis, thereby playing multifunctional roles in regulating cellular energetics, redox status, DNA methylation, and protein modification [30]. Therefore, we postulated that CBS would impact the trophoblast invasion process by altering the levels of MMPs 2, 9. In the current investigation, we assessed the expression of CBS and found that the plasma samples and placentae of EOPE patients had significantly lower CBS, MMP-2, and MMP-9 at both transcription and translation levels.

CBS has been reported to be expressed in a limited number of tissues with its gene promoters -1a and -1b coordinately regulated with proliferation through a redox-sensitive mechanism [10]. Studies in Drosophila SL2 cells have indicated that both promoters (-1a and -1b) are transactivated by specificity protein 1 (Sp1) and specificity protein 3 (Sp3) that form a ternary complex with each other before binding to the CBS -1b promoter region [10]. Interestingly, Sp1-deficient fibroblasts expressing both Sp3 and NF-Y showed no CBS activity, whereas rescue experiments with a mammalian Sp1 expression construct induced high levels of CBS activity, indicating that Sp1 has a critical and indispensable role in the regulation of CBS ^[10]^. In the current study, we observed significantly downregulated mRNA and protein expression of Sp1, likewise CBS, in EOPE patients, which could have led to the reduced levels of MMPs in these cases.

Sp1 has been identified as a novel and direct target of miR-22, using luciferase reporter assays [11]. A study in colorectal cancer cells reported diminished expression of Sp1 by miR-22 overexpression [11]. The study further reported that miR-22 could partially inverse Sp1-induced proliferation, colony formation, migration, and invasion in colorectal cancer cells. In the present study, we found an inverse pattern of expression of Sp1 and miR-22, i.e., downregulated gene expression of Sp1 and upregulated for that of miR-22 was observed in pregnancies complicated by EOPE as compared to normotensive, non-proteinuric controls. Shao et al. 2017 has reported enhanced miR-22 expression in EOPE placentae compared to their gestational age-matched preterm labor controls [12]. In the present study, we not only observed significantly upregulated levels of miR-22 (pre-miR-22 and miR-22-3p) in the placentae of EOPE patients but also in circulatory blood collected at diagnosis. 

The aberrant functioning of miR-22, Sp1, CBS, MMPs 2, 9, and TIMPs 1, 2 may be considered as one of the several putative factors present in patients' sera that not only contribute to various stresses but also reduce angiogenesis, migration, and invasion, thereby playing a significant role in contributing to maternal signs and symptoms of preeclampsia. 

The current study has significant limitations. It is a retrospective observational study with a limited sample size. Furthermore, because of the severity of the disease, in many cases, the pregnancy had to be ended quickly, which prevented additional laboratory testing. Lastly, the results may be affected by the fact that the hospital where the study was conducted is a tertiary referral center that treats the most severe cases.

## Conclusions

This is the very first study of its kind in which the down-regulation of MMPs 2, 9, CBS, and Sp1 and simultaneous upregulation of TIMPs 1, 2, and miR-22 (pre-miR-22 and miR-22-3p) expression/levels were observed in the EOPE patients, both in the placentae collected at delivery and the circulating blood collected at diagnosis. The MMPs 2, 9, CBS, Sp1 and miR-22 axis could presumably help explain the aetiology of the disease. The abnormal function of these markers may have contributed to the defective trophoblast invasion, a hallmark of PE, in the patients in our study population. The current study may assist in the development of a prediction algorithm for EOPE based on maternal circulatory factors coupled with imaging ultrasound risk predictors for designing future screening programs and planning preventive therapies for EOPE patients.

## Appendices

American College of Obstetricians and Gynecologists (ACOG) Guidelines

Blood Pressure- 140 mm Hg systolic or >90mm Hg diastolic on two occasions at least 4 hours apart after 20 weeks of gestational age in women with a previously normal BP and 160 mm Hg systolic or >110 mm Hg diastolic, confirmed within a short interval (minutes) to facilitate timely antihypertensive therapy; Proteinuria >300 mg per 24-hour urine collection or protein/ creatinine ratio > 0.3mg/dl or dipstick reading of 1+ or in the absence of proteinuria, new-onset hypertension with new onset of one or more of the following- thrombocytopenia: platelet count <100,000/μl, renal insufficiency: serum creatinine > 1.1mg/dl or doubling of serum creatinine in the absence of another renal disease, impaired liver function: elevated blood levels of liver transaminases to twice normal concentrations, pulmonary edema, and cerebral edema.
